# Estimating the economic burden of respiratory syncytial virus infections in infants in Vietnam: a cohort study

**DOI:** 10.1186/s12879-023-08024-2

**Published:** 2023-02-06

**Authors:** Lien Anh Ha Do, Elisabeth Vodicka, An Nguyen, Thi Ngoc Kim Le, Thi Thanh Hai Nguyen, Quang Tung Thai, Van Quang Pham, Thanh Uyen Pham, Thu Ngoc Nguyen, Kim Mulholland, Minh Thang Cao, Nguyen Thanh Nhan Le, Anh Tuan Tran, Clinton Pecenka

**Affiliations:** 1 New Vaccine Group, Murdoch Children’s Research Institute, 50 Flemington Road, Parkville, Melbourne, 3051 Australia; 2 Department of Pediatrics, The University of Melbourne, Melbourne, Australia; 3 PATH, Seattle, USA; 4 PATH, Hanoi, Vietnam; 5 Children’s Hospital 1, Ho Chi Minh City, Vietnam; 6 Pasteur Institute of Ho Chi Minh City, Ho Chi Minh City, Vietnam; 7 London School of Hygiene and Tropical Medicine, London, UK

**Keywords:** Respiratory syncytial virus, Lower respiratory tract infections, Direct cost, Indirect cost, Cost of illness

## Abstract

**Background:**

Little information is available on the costs of respiratory syncytial virus (RSV) in Vietnam or other low- and middle-income countries. Our study estimated the costs of LRTIs associated with RSV infection among children in southern Vietnam.

**Methods:**

We conducted a prospective cohort study evaluating household and societal costs associated with LRTIs stratified by RSV status and severity among children under 2 years old who sought care at a major pediatric referral hospital in southern Vietnam. Enrollment periods were September 2019–December 2019, October 2020–June 2021 and October 2021–December 2021. RSV status was confirmed by a validated RT-PCR assay. RSV rapid detection antigen (RDA) test performance was also evaluated. Data on resource utilization, direct medical and non-medical costs, and indirect costs were collected from billing records and supplemented by patient-level questionnaires. All costs are reported in 2022 US dollars.

**Results:**

536 children were enrolled in the study, with a median age of 7 months (interquartile range [IQR] 3–12). This included 210 (39.2%) children from the outpatient department, 318 children (59.3%) from the inpatient respiratory department (RD), and 8 children (1.5%) from the intensive care unit (ICU). Nearly 20% (105/536) were RSV positive: 3.9 percent (21/536) from the outpatient department, 15.7% (84/536) from the RD, and none from the ICU. The median total cost associated with LRTI per patient was US$52 (IQR 32–86) for outpatients and US$184 (IQR 109–287) for RD inpatients. For RSV-associated LRTIs, the median total cost per infection episode per patient was US$52 (IQR 32–85) for outpatients and US$165 (IQR 95–249) for RD inpatients. Total out-of-pocket costs of one non-ICU admission of RSV-associated LRTI ranged from 32%-70% of the monthly minimum wage per person (US$160) in Ho Chi Minh City. The sensitivity and the specificity of RSV RDA test were 88.2% (95% CI 63.6–98.5%) and 100% (95% CI 93.3–100%), respectively.

**Conclusion:**

These are the first data reporting the substantial economic burden of RSV-associated illness in young children in Vietnam. This study informs policymakers in planning health care resources and highlights the urgency of RSV disease prevention.

**Supplementary Information:**

The online version contains supplementary material available at 10.1186/s12879-023-08024-2.

## Background

Acute lower respiratory tract infections (LRTIs) are a major public health problem for children in Vietnam and in all low- and middle-income countries (LMICs). Respiratory syncytial virus (RSV) is the leading cause of LRTIs in pediatric populations. Epidemiological studies have consistently shown high rates of severe RSV infection in children between 1 and 5 months of age, with 99 percent of deaths occurring in LMICs where 90% of children under 5 years old live [1]. Two hospital-based studies in Ho Chi Minh City (HCMC), Vietnam, reinforce this: among children admitted with LRTIs, RSV prevalence ranged from 24 percent (children < 5 years) to 48 percent (children < 2 years) [2, 3]. A population-based LRTI surveillance site in central Vietnam also reported that RSV was the leading pathogen among children less than 2 years of age who were hospitalized with LRTIs [4].

The RSV season in South Vietnam typically occurs between May and November, during the rainy season [2–4]. Children’s Hospital 1 (CH1) in HCMC is a referral hospital for all provinces of South Vietnam. Bronchiolitis, commonly caused by RSV, has been the leading cause of hospitalization (averaging 49 percent of all-cause hospitalizations) among children less than 2 years of age, based on hospital records during the last 10 years. The average number of bronchiolitis admissions during the last 5 years was 6,956 children per year, two-thirds of whom were between 3 and 6 months of age. About 10 percent of those children were admitted to the emergency unit or intensive care unit (ICU) for oxygen supplementation, nasal continuous positive airways pressure (NCPAP), or other invasive respiratory assistance (CH1 records –unpublished data).

There is currently no licensed RSV vaccine. Passive prophylaxis for RSV is available through palivizumab (Synagis®, Astra Zeneca), a neutralizing anti-RSV fusion protein monoclonal antibody (mAb), but due to its high cost, palivizumab is not available or has minimal access in Vietnam and other LMICs [5].

Among the 40 recent RSV vaccine and mAb candidates, the long-acting mAb Nirsevimab™ (Sanofi, Astra Zeneca) is the most advanced and has shown encouraging results in Phase 2b and 3 trials [6]. Nirsevimab provides 50-fold greater activity and three-fold greater half-life (5 months protection [7]) compared to palivizumab. Nirsevimab requires just a single intramuscular injection for an entire RSV season and has been shown to reduce 70.1 to 78.4 percent of RSV-associated LRTIs in a Phase 2b trial [8, 9]. Nirsevimab was effective in preventing both RSV A and B subgroup infections. This mAb has been granted breakthrough therapy designation by the US Food and Drug Administration and Priority Medicine status by the European Medicines Agency [10]. Because of this, it could be available for clinical use soon—but it will likely be accessible only in high-income countries. In many countries, majority in LMICs, current treatment of RSV bronchiolitis includes a range of ineffective agents, such as systemic corticosteroids, bronchodilators, nebulized hypertonic saline, antibiotics, and anticholinergic agents. Supportive care (such as oxygen and respiratory support with high flow oxygen [NCPAP] or ventilation) are demonstrated to be effective [11], but are typically only accessible in settings with high skilled resources.

A robust estimate of RSV-related health burden and cost will help evaluate the potential impact and cost-effectiveness of future RSV prevention and inform national procurement and prioritization decisions. To our knowledge, no information is available on the costs of RSV in Vietnam and very little is available for other LMICs in Southeast Asia [12]. Although RSV was demonstrated to be the leading cause of LRTI hospitalizations, many pediatric hospitals in HCMC don’t have routine molecular RSV testing due to limited resources and the high cost of the molecular tests. A rapid detection antigen (RDA) test for RSV exists and would provide an alternative approach for hospitals that is lower cost than molecular testing, easy to use and has a high sensitivity and specificity; however, this has not yet been used widely in Vietnam. Early identification of RSV through a rapid test on admission would be helpful for patient management and treatments, particularly the prevention of nosocomial RSV infections, which are an important issue in Vietnam [13]. One commercially available RSV RDA test (Immuno AG1®, Fujifilm Corporation) is available for use in Japan and other places of European Union, but its real-world performance has not yet been evaluated against the gold standard molecular test in Vietnam.

We present here estimates of direct medical and non-medical costs and indirect costs of LRTIs associated with RSV infections among children visiting for consultations at outpatient departments or hospitalized at CH1. In this study, we also evaluated the performance of the RSV RDA test Immuno AG1 in a subset of patients.

## Methods

### Study setting

A prospective cohort study was conducted at the outpatient and inpatient departments of CH1, a tertiary pediatric care facility previously involved in studies assessing LRTIs and RSV in HCMC, Vietnam [3].

In general, patients with LRTIs, defined as cough or difficulty breathing [14], initially present to the outpatient department or emergency department at CH1. Those requiring additional services may be referred for inpatient care in the respiratory department or ICU, depending on the severity of LRTI disease. For the cost analysis, based on the clinical management, children with non-severe LRTI are those treated in the outpatient clinic; severe and very severe LRTI cases are typically treated in the respiratory department and in the ICU, respectively.

### Study population

We evaluated the costs associated with LRTIs, stratified by RSV status and severity, among children under 2 years of age seeking care at CH1.

Of note, the clinical definitions of RSV severity are not standardized and differ between settings. In our study we have adapted our definitions on severity groups from World Health Organization (WHO) recommendations [15] as below:

- Mild: patients admitted only to the outpatient department
- Moderate: patients admitted to the respiratory department and who had saturation oxygen (SaO_2_) at admission ≥ 93 percent
- Severe: patients admitted to the respiratory department and who had saturation oxygen at admission (SaO_2_) < 93 percent
- Very severe: patients admitted to ICU

Children were recruited to our study prospectively. Only patients who met all inclusion criteria and did not have any exclusion criteria were enrolled. Inclusion criteria were having cough and/or difficulty breathing, a clinical diagnosis of LRTI based on WHO criteria [14], and onset of symptoms ≤ 4 days prior to hospital admission. Exclusion criteria were patients with known non-respiratory or non-infectious respiratory diseases. Due to high volumes in the facility and to limit the burden on clinical staff and families, potential participants were identified and enrolled in the study if they had been hospitalized within 48 h and timing was convenient for the study doctors.

We used the formula below, based on WHO guidelines for estimating treatment costs for diarrheal disease [16], to calculate the minimum sample sizes for the primary objective of estimating the costs of RSV-associated LRTI cases in the (1) outpatient and (2) inpatient wards (stratified by severity–i.e., respiratory department and ICU):

$$n=ceiling {\left[\left(\frac{{precision}^{2}}{{CV}^{2} \times {Z}_{1-a/2}^{2}}+\frac{1}{{N}_{o}}\right)\right]}^{-1}$$

In the formula, CV is the coefficient of variation and N is the annual case load. Annual case loads for both outpatient and inpatient calculations were based on historical data from CH1 regarding annual LRTI admissions (N = 13,650 [3]). Data on visits to the outpatient ward were unavailable, so we assumed the same annual case load for outpatients and inpatients. While the outpatient ward sees more cases of LRTI than the inpatient setting, outpatient cases are less severe and will have less variation in resource needs and costs than inpatient cases. We assumed a Z-score of 1.96 (95 percent confidence interval [CI]), margin of error of 10 percent, and coefficient of variation of 0.5. This yielded target sample sizes of 96 (rounded up to 100) and 139 (rounded up to 150) RSV-positive children in the outpatient and inpatient settings, respectively. To estimate the number needed to achieve the desired sample sizes, we assumed an RSV-positive rate of 30 to 50 percent based on previous RSV studies at CH1 [3]. This determined we needed to enroll 200 to 330 outpatients and 300 to 500 inpatients, depending on observed prevalence during the RSV season.

### Study timeline

The study began in September 2019 with three enrollment periods: September 2019–December 2019, October 2020–June 2021 and October 2021–December 2021. Due to COVID-19 pandemic, the study had to pause for two periods from January to September 2020 and from July to September 2021.

### Sample collection and RSV detection

RSV testing is not routine at CH1. A nasopharyngeal (NP) swab was collected from each study participant, within 24 h of study enrollment, to determine RSV infection status using a validated real-time RT-PCR (reverse transcription polymerase chain reaction) test [17]. NPs for RT-PCR testing were collected in RNAlater™ media (Invitrogen, Lithuania) and transported to the Pasteur Institute laboratory on wet ice within 24 h of collection where they were stored at − 80 °C until RT-PCR testing performed at the end of each study enrolment period.

A random sample of 30 percent of participants admitted to the respiratory ward department also had an additional NP swab (collected at the same time of the NP swab collection for the real-time RT-PCR). This additional NP swab was tested immediately on-site via a commercial RSV RDA test (Immuno AG1®, Fujifilm Corporation).

Although the physicians who treated patients were not aware of RSV RT-PCR results, they received results from the RDA test in real-time. All laboratory technicians who conducted the RT-PCR testing were blinded about the RSV RDA results.

### Data collection and components

Costs were estimated from the societal and household perspectives. Three components of cost and resource utilization data were collected for the study:

(1) Health care utilization and cost data from facility-level billing data.

(2) A day-of-visit parent questionnaire to assess household-level costs incurred prior to arriving at the facility. For outpatient participants, any costs due to seeking care incurred on the day of the visit were also collected.

(3) A post-discharge parent questionnaire to identify additional costs incurred by the household for the same illness 1 week after discharge from the facility.

The billing data were used to identify resource utilization as well as direct medical and overhead costs associated with care received in the facility. Data parameters collected include types of services received during the visit (ICU bed, inpatient bed, outpatient services), consultation fees, medications, laboratory tests, imaging (chest X-ray, echocardiogram, ultrasound), and other procedures (chest physiotherapy, intubations, oxygen delivery). The proportion of costs covered by the household and national health insurance system (NHIS) was also reflected on facility-level billing data. Consumption of health care was the direct medical cost that covered the total costs of disease episode.

Hence, we reported:

- The total costs per case from the societal perspective including direct medical costs (from both billing and questionnaire data), non-medical costs, and indirect costs incurred prior to admission, during the visit and after discharge.
- The household-level costs included patient-level out-of-pocket, non-medical costs (e.g., transportation to/from facility, meals, accommodations, and caregiver), and indirect costs (e.g., opportunity costs of lost income and lost leisure time) during the disease course. These costs were obtained through standardized questionnaires conducted with parents (pre- and post-discharge), as described above.

We also collected data on total costs, stratified by time of spending, using a specific questionnaire for each spending period. Pre-hospital costs include direct medical costs, non-medical costs, and indirect costs estimated from household surveys for any health care services sought before the day of outpatient visit or inpatient/ICU admission. Costs incurred during the hospital stay include direct medical costs from billing data plus non-medical and medical out-of-pocket spending (beyond the hospital charge) for the day of outpatient visit or the episode of inpatient stay, as well as indirect costs incurred during the disease episode. Similarly, follow-up visit costs include direct medical costs from billing data for follow-up visits at the hospital, as well as out-of-pocket spending and indirect costs for any care sought after the day of outpatient visit or discharge until completing a follow-up visit.

The NHIS covers a certain proportion of the direct medical cost, depending on the type of medicines and procedures received during the hospitalization or the visit. The NHIS coverage amount was reflected on the billing data collected for the study. The remaining cost was from the out-of-pocket expenditures by the patient’s family (e.g., the household-level cost).

Demographic characteristics of children and their families were also collected through questionnaires for both outpatients and inpatients.

**Fig. 1:**
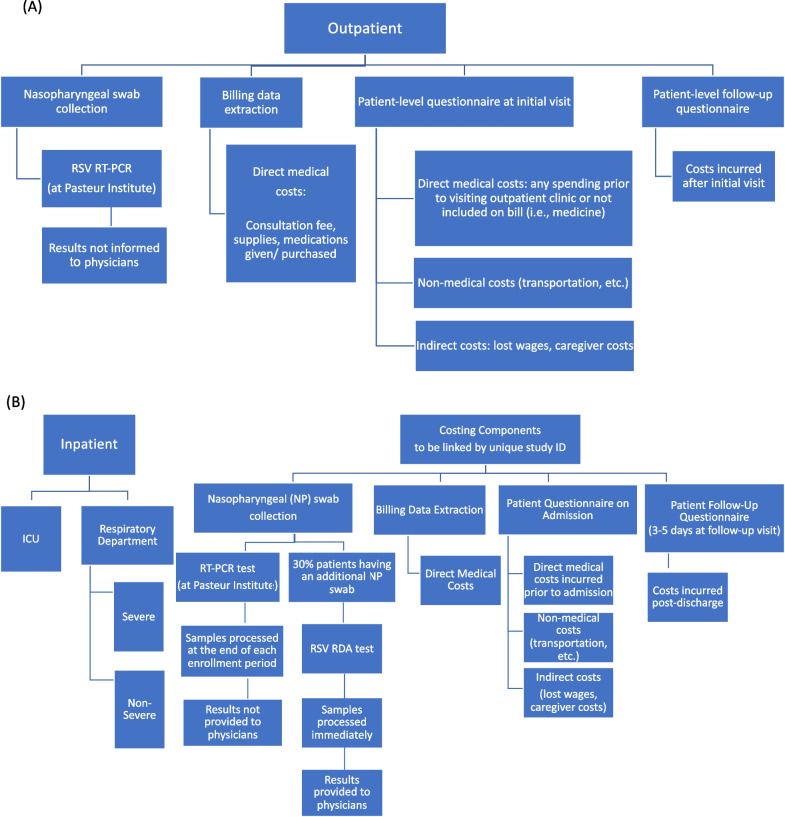
Clinical flow process and the data components collected from outpatients and inpatients. **A** Flowchart of outpatient study components and data elements to be collected. **B** Flowchart of inpatient study components and data elements to be collected

Figure 1 represents the clinical flow process and data components collected from outpatients and inpatients.

### Data analysis

Costs were estimated by the following categories: cost per episode, cost by component (i.e., direct medical costs, direct non-medical costs, indirect costs, and relevant subcategories), and cost by RSV status and severity (outpatient, respiratory department, ICU).

In calculating indirect costs, opportunity costs of lost income due to missed work were based on the average salary reported by participants in the study, and opportunity costs of lost leisure time were based on the minimum Vietnamese income based on government data, for the study period [18]. Continuous variables were presented as the median, with interquartile ranges (IQRs) of 25 to 75 percent. Categorical variables were presented as numbers and percentages. We used the July 2022 exchange rate of 1 US dollar for 23,195 Vietnam Dong (VND) to convert VND costs to US dollars [19].

We also evaluated sensitivity, specificity, and positive and negative predictive value of the RSV antigen rapid test (compared to the validated real-time RT-PCR) and calculated 95 percent CIs. Statistical comparisons between the RSV antigen rapid test and the validated real-time RT-PCR were performed using McNemar’s test [20].

All statistical analysis was implemented using Stata version 16.0 (StataCorp LP). All statistical tests were conducted at the two-tailed 5 percent significance level.

## Results

### Study population characteristics

We enrolled 537 children in our study. One case was discarded from the final analysis as more than 50 percent of the relevant data were missing, including the billing cost and the questionnaire data; thus, the full analysis was conducted on 536 participants. This included 210 children (39.2 percent) from the outpatient department; 318 children (59.3 percent) from the respiratory department; and 8 children (1.5 percent) from the ICU. Demographic and clinical characteristics of patients are shown in Table 1. Median age was lowest for patients from the ICU and highest for patients from the outpatient department. The median duration of hospitalization for ICU patients was three times higher than for those in the respiratory department (Table 1). Guardians or parents of children receiving care in the ICU had less educational background compared to those in the outpatient and respiratory departments (Table 1). The majority of ICU patients (75 percent) were from outlying provinces. In terms of RSV detection, 103/536 (19.21 percent) were RSV positive: 21 patients were from the outpatient department and 84 from the respiratory department. RSV was not detected among the 8 ICU cases. No deaths were recorded from our study participants.

**Table 1:** Demographic and clinical characteristics of patients

Characteristics	Outpatients			Inpatients at RD			ICU^**^
	Total (N = 210)	RSV positive (n = 21)	RSV negative (n = 189)	Total (N = 318)	RSV positive (n = 84)	RSV negative (n = 234)	Total (n = 8)
Age in months, median (IQR)	8 (5–14)	8 (6–17)	8 (5–14)	6 (2–11)	3 (2–11)	6 (2–11)	2.5 (1–3.5)
Number of females, n (%)	76 (36.2)	8 (38.1)	68 (36.0)	138 (43.5)	40 (47.6)	98 (42.1)	3 (37.5)
Number in HCMC, n (%)	104 (49.5)	12 (57.1)	92 (48.7)	123 (38.8)	30 (35.7)	93 (39.7)	2 (25.0)
Number of people living in household, median (IQR)	4 (4–6)	4 (4–6)	4 (4–6)	4 (4–5)	4 (4–5)	4 (4–5)	4 (4–5.5)
Number of children under 5 years old living in household, median (IQR)	1 (1–2)	1 (1–1)	1 (1–2)	1 (1–2)	1 (1–2)	1 (1–2)	1 (1–2.5)
Primary caregiver education of high school or higher, n (%)	96/170 (56.5)	7/15 (46.7)	89/155 (57.4)	199/313 (63.6)	52 (61.9)	147 (64.2)	2 (25.0)
Diagnosis at admission							
Bronchiolitis, n (%)	116 (55.2)	19 (90.5)	97 (51.3)	24 (7.6)	11 (13.1)	13 (5.6)	0 (0)
Pneumonia, n (%)	3 (1.4)	0 (0)	3 (1.6)	106 (33.4)	30 (35.7)	76 (32.6)	8 (100)
Other LRTIs, n (%)	88 (41.9)	2 (9.5)	86 (45.5)	186 (58.7)	43 (51.2)	143 (61.4)	0 (0)
Oxygen saturation (SaO_2_)*							
SaO_2_ < 90%, n (%)	N/A	N/A	N/A	3 (0.9)	2 (2.4)	1 (0.4)	1 (12.5)
SaO_2_ 90–92	N/A	N/A	N/A	5 (1.6)	4 (4.8)	1 (0.4)	2 (25.0)
SaO_2_ 93 or above	N/A	N/A	N/A	177 (56.2)	70 (83.3)	107 (46.3)	5(62.5)
Duration of hospitalization in days, median (IQR)	N/A	N/A	N/A	7 (6–9)	7 (6–8)	7 (6–9)	24 (11.5–49.5)

^*^Oxygen saturation (SaO_2_) was measured at admission as a marker of the severity of LRTIs (see method section)

^**^No ICU cases were identified as RSV positive

HCMC: Ho Chi Minh City; ICU: intensive care unit; IQR: interquartile range; LRTI: lower respiratory tract infection; RD: Respiratory Department; RSV: respiratory syncytial virus

### Societal costs

The median total cost from the societal perspective per LRTI episode (regardless of RSV status) per patient was US$52 (IQR 32–86) for outpatients and US$184 (IQR 110–287) for inpatients (not including ICU patients). As the costs of ICU cases were substantial compared to the costs of other inpatients, we estimated the total costs of ICU cases separately. The total cost per ICU admission was an 11-times the total cost per non-ICU admission (Table 2).

**Table 2:** Cost associated with the societal perspective (median, IQR), in 2022 US dollars

Cost components	Outpatient				Inpatient at RD				ICU^***^
	All (N = 210)	RSV positive (n = 21)	RSV negative (n = 189)	p-value^**^	All inpatients (N = 318)	RSV positive (n = 84)	RSV Negative (n = 234)	p-value^**^	All RSV negative (n = 8)
Total costs	52 (32–86)	52 (32–85)	52 (3 2–86)	0.71	184 (110–287)	165 (95–249)	190 (123–292)	0.04	2146 (1038–4067)
Direct medical costs	22 (13–32)	26 (16–32)	21 (13–32)	0.35	84 (50–135)	68 (47–102)	91 (59–153)	0.001	1996 (937–3786)
Consultation fee	3 (2–4)	2 (2–3)	3 (2–4)	0.20	3 (2–6)	3 (2–6)	3 (2–5)	0.67	4 (2–6)
Medication costs	15 (9–20)	15 (8–18)	15 (9–20)	0.46	9 (4–19)	10 (4–21)	9 (5–19)	0.85	114 (20–678)
Laboratory costs	0 (0–2)	0 (0–2)	0 (0–2)	0.88	2 (2–13)	2 (0–3)	4 (2–17)	< 0.001	193 (64–359)
Procedure & service cost	6 (3–11)	8 (4–13)	6 (3–11)	0.14	49 (24–71)	29 (19–58)	50 (29–88)	< 0.001	1356 (524–2468)
Imaging costs	3 (3–5)	3 (3–3)	3 (3–5)	0.85	6 (3–16)	3 (3–12)	6 (3–16)	0.03	28 (10–85)
Miscellaneous costs*	8 (1–13)	0 (0–0)	8 (1–13)	N/A	13 (6–23)	12 (6–23)	13 (6–23)	0.31	204 (120–332)
Direct non-medical costs	5 (2–28)	4 (2–22)	5 (2–28)	0.82	35 (3–87)	27 (3–72)	38 (3–88)	0.57	76 (42–112)
Transport costs	4 (2–27)	4 (2–22)	4 (2–27)	0.91	6 (2–28)	8 (2–41)	5 (1–24)	0.12	33 (3–78)
Meal costs	0 (00)	0 (0–0)	0 (0–0)	0.18	32 (0–63)	2 (0–39)	41 (0–70)	0.001	5 (2–52)
Accommodation costs	0 (0–0)	0 (0–0)	0 (0–0)	N/A	0 (0–30)	0 (0–0)	16 (0–32)	< 0.001	10 (0–233)
Caretaker costs	0 (0–0)	0 (0–0)	0 (0–0)	N/A	6 (0–38)	47 (34–60)	0 (0–13)	0.07	0 (0–0)
Indirect costs	11 (2–26)	22 (9–34)	9 (0–25)	0.09	19 (0–69)	18 (0–69)	20 (0–69)	0.93	85 (6–253)
Lost income	9 (0–25)	17 (9–34)	8 (0–22)	0.03	17 (0–69)	12 (0–68)	17 (0–69)	0.85	13 (4–194)
Lost leisure	6 (2–6)	3 (0–6)	6 (2–6)	0.18	2 (1–6)	6 (2–9)	2 (1–5)	0.05	3 (2–5)
Prior to hospital costs	11 (1–26)	5 (1–22)	11 (1–26)	0.43	17 (9–35)	17 (9–37)	18 (9–35)	0.82	45 (25–93)
In-hospital costs	29 (17–49)	27 (11–35)	30 (17–49)	0.32	161 (94–268)	137 (72–214)	181 (103–282)	0.005	1660 (861–4010)
Follow-up visit costs	18 (7–33)	26 (16–49)	17 (7–32)	0.05	0 (0–9)	0 (0–6)	2 (0–11)	0.005	13 (2–227)

^*^Miscellaneous costs include medical consumables such as syringes, gloves, and soap

^**^Mann–Whitney U p-value comparing between the costs associated with RSV and those associated with no RSV

^***^No ICU cases were identified as RSV positive

ICU: intensive care unit; IQR: interquartile range; RD: Respiratory Department; RSV: respiratory syncytial virus, N/A: not applicable

For RSV-associated LRTI cases, the median total cost was US$52 (IQR 32–85) for outpatients and US$165 (IQR 95–249) for inpatients (Table 2). Direct medical costs accounted for the highest proportion of the total cost for both RSV outpatient and inpatients, which were 40 percent and 48 percent, respectively. Among RSV outpatients, non-medical costs (e.g., transportation, accommodations, etc.) and indirect costs (e.g., the opportunity cost of missed work) contributed 9 percent and 40 percent of the total cost. Among RSV inpatients, non-medical costs and indirect costs contributed 18 percent and 14 percent of the total cost.

For non-RSV LRTI cases, the median total cost per disease episode per patient was US$52 (IQR 32–86) for outpatients and US$190 (IQR 123–292) for inpatients. The median total cost per disease episode for RSV-negative cases was significantly higher than the total cost of RSV-positive cases (Mann–Whitney U p-value = 0.04) for inpatients. Specifically, the direct medical cost, in-hospital cost, and follow-up cost for non-RSV LRTI cases were significantly higher than those for RSV-LRTI cases (Table 2) (Mann–Whitney U p-value = 0.001, 0.005 and 0.005, respectively). RSV-LRTI cases reported more lost leisure time than the non-RSV-LRTI cases for inpatients (Mann–Whitney U p-value = 0.05) (Table 2).

### Household costs

Approximately 1 percent (3/210) and 86.8 percent (276/318) of outpatients and inpatients were covered by the NHIS, respectively.

The median total household cost per LRTI period per patient was US$51 (IQR 32–85) for outpatients and US$112 (IQR 58–184) for inpatients (not including ICU patients). The total household cost per ICU admission was a 5-times the total household cost per non-ICU admission (Table 3).

**Table 3:** Household-level out-of-pocket costs and payment sources (median, IQR), in 2022 US dollars

Cost components	Outpatient				Inpatient at RD				ICU****
	All (N = 210)	RSV positive (n = 21)	RSV negative (n = 189)	p-value^***^	All (N = 318)	RSV positive (n = 84)	RSV negative (n = 234)	p-value^***^	All RSV negatives (n = 8)
Total out-of-pocket costs	51 (32–85)	52 (32–85)	51 (32–84)	0.71	112 (58–184)	117 (65–183)	110 (57–186)	0.45	543 (370–996)
(1) Direct medical costs	22 (13–32)	26 (16–32)	21 (12–32)	0.33	24 (13–49)	38 (20–61)	20 (12–37)	< 0.001	386 (161–813)
Consultation fee	3 (2–4)	2 (2–3)	3 (2–4)	0.20	3 (2–6)	3 (2–6)	3 (2–5)	0.66	4 (2–6)
Medication costs	15 (9–20)	15 (8–18)	15 (9–20)	0.49	3 (0–9)	6 (1–13)	2 (0–6)	< 0.001	33 (13–62)
Laboratory costs	0 (0–2)	0 (0–2)	0 (0–0)	0.81	0 (0–2)	1 (0–3)	0 (0–2)	0.52	7 (0–45)
Procedure & service cost	6 (3–11)	8 (4–13)	4 (3–11)	0.12	2 (0–16)	12 (6–23)	0 (0–13)	< 0.001	86 (30–311)
Imaging costs	3 (3–5)	3 (3–3)	3 (3–5)	0.87	0 (0–3)	2 (1–5)	0 (0–2)	< 0.001	0 (0–4)
Miscellaneous costs*	8 (1–13)	0 (0–0)	8 (1–13)	–	9 (3–18)	10 (4–18)	9 (2–18)	0.42	105 (79–158)
(2) Direct non-medical costs	5 (2–28)	4 (2–22)	5 (2–28)	0.82	35 (3–87)	27 (3–72)	38 (3–88)	0.57	76 (42–112)
Transport costs	4 (2–27)	4 (2–22)	4 (2–27)	0.91	6 (2–28)	8 (2–41)	5 (1–24)	0.12	33 (3–78)
Meal costs	0 (0–0)	0 (0–0)	0 (0–0)	0.18	32 (0–63)	2 (0–39)	41 (0–70)	< 0.001	5 (2–52)
Accommodation costs	0 (0–0)	0 (0–0)	0 (0–0)	N/A	0 (0–30)	0 (0–0)	16 (0–32)	< 0.001	10 (0–233)
Caretaker costs	0 (0–0)	0 (0–0)	0 (0–0)	N/A	6 (0–38)	47 (34–60)	0 (0–13)	0.08	0 (0–0)
(3) Indirect costs	11 (2–26)	22 (9–34)	9 (0–25)	0.09	19 (0–69)	18 (0–69)	20 (0–69)	0.93	85 (6–253)
Lost income**	9 (0–25)	17 (9–34)	8 (0–22)	0.03	17 (0–69)	12 (0–68)	17 (0–69)	0.85	13 (4–194)
Lost leisure**	6 (2–6)	3 (0–6)	6 (2–6)	0.18	2 (1–6)	6 (2–9)	2 (1–5)	0.05	3 (2–5)
Family response									
Reducing other expenses	34.3%	9.5%	37.0%	0.01	59.0%	42.9%	64.8%	< 0.001	25.0%
Use personal savings	87.1%	100.0%	85.7%	0.06	86.8%	94.1%	84.1%	0.02	100.0%
Borrowed	2.4%	0.0%	2.7%	0.45	26.5%	6.0%	33.9%	< 0.001	12.5%
Selling assets	2.4%	4.8%	2.1%	0.45	5.7%	2.4%	6.9%	0.13	0.0%
Donations	2.9%	0.0%	3.2%	0.41	16.4%	3.6%	21.0%	< 0.001	0.0%
Other	0%	0.0%	0.0%	N/A	0.3%	0.0%	0.4%	0.55	0.0%

Total out-of-pocket costs = sum of (1–3). These costs were incurred prior to, during, and after hospitalization. (2) and (3) are the same as costs associated with the societal perspective in Table 2

^*^Miscellaneous costs include medical consumables such as syringes, gloves, and soap

^**^Opportunity costs of income lost due to missed work were based on the average salary reported by participants in the study, and opportunity costs of lost leisure time were based on the minimum Vietnamese income based on government data for the study period [18]

^***^Mann–Whitney U* p*-value comparing between the costs associated with RSV and those associated with no RSV

^****^No ICU cases were identified as RSV positive

ICU: intensive care unit; IQR: interquartile range; RD: Respiratory Department; RSV: respiratory syncytial virus

Most families of outpatients (87.1 percent), non-ICU inpatients (86.8 percent), and ICU patients (100 percent) reported having to use their savings to cover disease treatment and lost income (Table 3).

For RSV-associated LRTI cases, the median total household cost per infection per patient was US$52 (IQR 32–85) for outpatients and US$117 (IQR 65–183) for inpatients (Table 3).

### Consumption of health care

Details of what was included in the direct medical cost per disease episode for outpatients and inpatients are presented in Table 4. The median direct medical costs per disease episode for outpatients and inpatients were US$22 (IQR 13–32) and US$84 (IQR 50–135), respectively. The total direct medical cost per ICU admission was about 24 times higher than the total direct medical cost per non-ICU admission (Table 4). The cost by RSV status was also presented for outpatient and inpatient groups (with the exception of patients from the ICU, who were all negative for RSV).

**Table 4:** Detailed breakdown of direct medical cost components (median, IQR), in 2022 US dollars

Cost components	Outpatients				Inpatients at RD				ICU
	All (N = 210)	RSV positive (n = 21)	RSV negative (n = 189)	p-value^**^	All (N = 318)	RSV positive (n = 84)	RSV negative (n = 234)	p-value^**^	All RSV negative^***^ (n = 8)
Total direct medical costs	22 (13–32)	26 (16–32)	21 (13–32)	0.35	84 (50–135)	68 (47–102)	91 (59–153)	0.001	1996 (937–3786)
Medication costs	15 (9–20)	15 (8–18)	15 (9–20)	0.46	9 (4–19)	10 (4–21)	9 (5–19)	0.85	114 (20–678)
Antibiotics	3 (2–5)	2 (1–4)	3 (2–5)	0.06	2 (1–4)	1 (1–3)	2 (1–5)	0.003	82 (2–705)
Corticosteroids	1 (0–1)	0 (0–0)	1 (0–1)	0.16	0 (0–0)	0 (0–1)	0 (0–0)	0.33	0 (0–17)
Bronchodilator oral	1 (1–1)	1 (1–1)	1 (1–1)	0.16	1 (1–1)	1 (0–1)	1 (1–1)	0.95	0 (0–0)
Bronchodilator inhalation	1 (1–3)	2 (1–3)	1 (1–3)	0.94	5 (2–18)	11 (2–18)	4 (2–17)	0.48	7 (2–13)
Other drugs	4 (3–6)	4 (3–5)	4 (3–7)	0.25	2 (2–4)	2 (2–3)	2 (2–4)	0.04	52 (11–367)
Laboratory costs	0 (0–2)	0 (0–2)	0 (0–2)	0.88	2 (2–13)	2 (0–3)	4 (2–17)	< 0.001	193 (64–359)
Imaging costs	3 (3–5)	3 (3–3)	3 (3–5)	0.85	6 (3–16)	3 (3–12)	6 (3–16)	0.03	28 (10–85)
Chest X-ray	3 (3–3)	3 (3–3)	3 (3–3)	0.80	3 (3–6)	3 (3–3)	3 (3–6)	0.37	6 (4–11)
Echocardiogram	9 (9–9)	9 (9–9)	9 (9–9)	N/A	7 (1–12)	0 (0–0)	7 (1–12)	N/A	3 (3–3)
Ultrasound	2 (2–9)	2 (2–2)	2 (2–9)	0.43	13 (2–13)	2 (2–4)	13 (4–13)	< 0.001	8 (3–10)
Procedure & service costs	6 (3–11)	8 (4–13)	6 (3–11)	0.14	49 (24–71)	29 (19–58)	50 (29–88)	< 0.001	1,356 (524–2468)
Consultation costs	3 (2–4)	2 (2–3)	3 (2–4)	0.20	3 (2–6)	3 (2–6)	3 (2–5)	0.67	4 (2–6)
Miscellaneous costs*	8 (1–13)	0 (0–0)	8 (1–3)	N/A	13 (6–23)	12 (6–23)	13 (6–23)	0.31	204 (120–332)

^*^Miscellaneous costs include medical consumables such as syringes, gloves, and soap

^**^Mann–Whitney U p-value comparing between the costs associated with RSV and those associated with no RSV

^***^No ICU cases were identified as RSV positive

ICU: intensive care unit; IQR: interquartile range; RD: Respiratory Department; RSV: respiratory syncytial virus; N/A: not applicable

Medication and procedure costs represented the highest portion of the medical costs among LRTIs in outpatients and inpatients, including ICU patients (Table 4). The procedure costs included any medical interventions (such as oxygen delivery through nasal canal or facial mask, intubation or chest physiotherapy/breathing exercises, and the cost of the hospital bed).

Among outpatients, RSV treatment costs were not significantly different compared to the non-RSV LRTI costs (Mann–Whitney U p-value = 0.39). Among inpatients, significant differences in the antibiotic, laboratory cost, imaging and procedure costs were observed between the RSV and non-RSV groups (Mann–Whitney U p-value = 0.003, 0.000, 0.03 and 0.001, respectively) (Table 4). Non-RSV cases required more antibiotics, laboratory test, imaging, and procedures than RSV cases.

### Sensitivity and specificity of RSV rapid detection antigen (RDA)

In total, we had 70 cases tested by both RSV RDA and RSV real-time RT-PCR. The number of RSV detections by RSV RDA test (Immuno AG1) test was not significantly lower than by real-time RT-PCR (15/70 [21.4 percent] versus 17/70 [24.3 percent), McNemar test p-value = 0.5). Table 5 showed the number of positive and negative cases identified by RSV RDA test (Immuno AG1) test or by RSV real-time RT-PCR.

**Table 5:** Sensitivity and specificity of RSV RDA test (Immuno AG1)

		RSV real-time RT-PCR		
		Positive	Negative	Total
RSV RDA test (Immuno AG1)	Positive	15	0	15
	Negative	2	53	55
	Total	17	53	70

RT-PCR: reverse transcription polymerase chain reaction; RSV: respiratory syncytial virus

Based on the results shown in Table 5, the sensitivity of the RSV RDA test was 15/17 (88.2 percent, 95 percent CI 63.6–98.5 percent) and the specificity was 53/53 (100 percent, 95 percent CI 93.3–100 percent). Positive predictive value and negative predictive value of the RSV antigen rapid test were 15/15 (100 percent, 95 percent CI 78.2–100 percent) and 53/55 (96.3 percent, 95 percent CI 87.5–99.6 percent).

## Discussion

Our study showed the costs associated with LRTIs are substantial and somewhat higher among children with RSV-negative status and increased severity of disease. For outpatient visits, a patient’s family usually paid out-of-pocket for examination and treatment. Inpatients less than 5 years of age are covered by the NHIS at 80 percent of the costs of listed medications and interventions if they follow their catchment area for hospitalization, are admitted due to an emergency, or are formally referred to a tertiary public hospital. Despite this coverage, the total out-of-pocket cost for outpatients and non-ICU inpatients for one LRTI event were still significant, representing about 32 to 70 percent of the monthly minimum wage per person ($160) [18] in HCMC. In addition to the immediate economic impact on households, the cost of ICU hospitalization could result in long-term debt for the patient’s family, as 100 percent of families in our study reported having to use their savings to pay for care. More than 12 percent of families had to borrow money to support their child’s hospital stay (Table 3).

During the first year of life, incidences of acute lower respiratory infections (ALRI) leading to outpatient visit and hospitalization were previously reported to be 461 and 81 per 1000 children, respectively [21]. Among children with ALRI in the birth cohort, 24 outpatient visits and seven hospitalizations were associated with RSV [21]. For children living in provinces, these incidences of outpatient visits and hospitalizations were about five- and two-times higher compared to those in HCMC, respectively [21]. These incidences have highlighted the substantial economic burden of LRTIs generally, including those associated with RSV infection. Although the number of ICU cases in our study was small, all cases occurred under 3 months of age, whereas the median ages were 6 and 8 months among inpatients and outpatients, respectively. These data support previous studies that demonstrated young infants are particularly vulnerable to severe LRTIs [2, 3].

Because the clinicians in our study were not blinded for the RSV RDA result, this could have influenced their decisions on the treatment and management for those cases. We compared the demographics, clinical characteristics, societal costs, out-of-pocket costs and the consumption of health care system costs between patients having their sample processed with the RDA test and those who did not have the RDA test (Additional file 1: Tables S1, S2, S3 and S4). We found significant differences in the societal and health care system costs between the two groups (Mann–Whitney U p-value = 0.009 and 0.016, respectively). These differences can be explained by the difference in LRTI disease severity, where the RDA tested group had significantly more pneumonia than the non-RDA tested group [32/70 (45.7%) versus 74/248 (29.8%), Chi-square p-value = 0.013]. This can also be explained by the fact that clinicians using the RDA test ordered more investigations for those who were known to be RSV-negative, making the median direct medical cost of the whole RDA tested group higher. In fact, in the RDA tested group, 53 RSV negative cases had significantly higher direct medical costs compared to 17 RSV positive cases [median cost US$60 (IQR 36–75) versus US$19 (IQR 12–29), Mann–Whitney U p-value = 0.001]. Despite the differences in resource utilization by RSV test type, our study is unable to determine whether the presence of immediate results influenced clinical behavior or costs, as it was outside the scope of our study design. Interestingly, although clinicians were not informed about the RSV positivity results for the remaining 248 cases that were tested only with the RT-PCR, a similar pattern was observed with the patients with non-RSV LRTI receiving more imaging tests, and thus yielding a higher total cost for imaging. The significantly higher total costs of non-RSV LRTI cases compared to RSV LRTI cases (p = 0.03) could also be explained by the fact that more non-RSV LRTIs occurred in patients traveling from outside HCMC to seek care, leading to greater non-medical costs such as meals and accommodations (although differences in these costs were not statistically significant when compared to the RSV LRTIs group). Due to the limited number of health care staff in Vietnam, the hospitals mainly take care of medical treatments and rely on patients’ families to provide everyday “nursing tasks.” This requires the family of patients who come from provinces to stay in or near the hospitals 24/7, leading to significant meal and accommodation expenses. For example, a previous study in Hanoi, Vietnam, showed that household members made an average of 26 visits to a pneumonia patient who stayed in hospitals for 7 days on average [22]. Meals and accommodation cost in our study represent more than 50 percent of direct non-medical costs in the non-RSV LRTIs group (Table 2).

In our study, medical interventions during the hospital stay in LRTI patients account for the largest portion of direct medical costs. The clinical impact and cost-effectiveness of chest physiotherapy, which was a frequently reported procedure in our patients, is still debated [23]. Differences in clinical management of LRTIs in general and of RSV-LRTIs specifically have been observed between hospitals in Vietnam and between countries [22, 24, 25]. This includes the testing given, types of antibiotics prescribed, and dosing schedules, as well as the threshold for hospital admission, all of which have an impact on the direct medical cost. These differences also affect the duration of hospitalization, which heavily impact cost. The direct medical cost of LRTIs in our study is quite similar to the reported cost of pneumonia in Khanh Hoa province in Vietnam, but much lower than the cost reported in Bach Mai hospital [22, 25] as well as in the modelling estimation by Li et al. [26]. We recognize the limitations of generalizability of our cost estimation, which may not be representative of resource utilization and costs across provinces in Vietnam nationally. CH1 is considered the primary public pediatric hospital in Ho Chi Minh City and receives patients from Central to South Vietnam. In that respect it can be expected that this is a reasonable representation of care provided in Vietnam.

In our study, other pathogens (non-RSV status) and severity were the two factors significantly associated with the total costs (Table 2), specifically with the direct medical cost. This highlights the importance of the pathogen etiology context of a study population, as 23.9 to 24.5 percent of children under 2 years of age in Vietnam may carry *Streptococcus (S.) pneumoniae* [27]. RSV co-infection with *S. pneumoniae* carriage has been previously reported to significantly increase the *S. pneumoniae* load and be associated with radiologically confirmed pneumonia [28], which leads to longer hospitalizations and higher disease costs. There is still potentially high prevalence of pneumococcal diseases in Vietnam because pneumococcal conjugate vaccines (PCVs) have not yet been introduced in the Vietnam National Immunization Program due to their high cost. PCVs are available only through out-of-pocket expenditure [29].

Of note, it has been shown that the incidences of severe RSV cases and associated deaths have been disproportionally reported in LMICs such as Vietnam [30]. The high burden of LRTIs observed in Vietnam [2–4] leads not only to a high expense in direct medical care but also disrupts the workforce, as seen through the indirect costs that include the missed days of work due to seeking care. This causes a vicious “disease-economic” cycle suffered by many families in LMICs.

There are some limitations in our study that should be acknowledged. First, our study was conducted during the COVID-19 pandemic, limiting the patient enrollment and interrupting data collection. The usual RSV season also changed during COVID-19 and became hard to predict. It was not clear if RSV infections during COVID-19 pandemic are less severe than pre-pandemic infections. We did not see differences in severity but cannot make conclusions as our sample size was small. Although we met our lower-bound target for the number of children to be enrolled (based on an expected 30 percent RSV-positivity rate), the overall number of RSV cases in our study was lower than anticipated (19 percent) and, thus, we did not reach the estimated sample sizes for RSV-positive cases, limiting the certainty of these cost estimates. Second, we did not have RSV cases among ICU patients. We were able to characterize non-RSV ICU costs, but we cannot draw concrete costs for very severe RSV cases. Hence, this study does not allow us to describe the whole spectrum of costs associated with RSV disease. Third, we did not record information about number of eligible patients across the facility due to high hospital volumes and, therefore, are not able to characterize the difference between eligible LRTI patients and those enrolled in our study. This limits our ability to evaluate potential selection bias and generalizability of the study. Fourth, our costs relied on billing data, which often reflect charges to the patient or payer and may or may not reflect the true costs of resource utilization. Finally, we did not have the information about the gestational age of patients in our study, we cannot estimate what the impacts of the preterm births on RSV cost. Compared to term infants, preterm infants have a 2- to threefold higher hospitalisation rate [31, 32] and fivefold higher mortality rate [33]. Pre-term population can cause a significant cost on the health care system.

## Conclusion

This study provides, for the first time, the complete costs (direct and indirect) associated with LRTIs in Vietnam in general and with RSV-LRTIs specifically. These costs are substantial, leading to significant economic burden to families and potentially to the health care system and country as the NHIS covers about 80 percent of the direct medical cost. These cost estimates provide valuable data for economic analysis of LRTI management and preventive strategies generally and for LRTIs associated with RSV.

## Supplementary Information

**Additional file 1. **Additional Tables.

## Data Availability

The datasets generated and/or analysed during the current study are not publicly available due to restrictions on data use specified by project partners but are available from the corresponding author on reasonable request.

## Abbreviations

ALRI: Acute lower respiratory infections
CH1: Children’s Hospital 1
CI: Confidence interval
HCMC: Ho Chi Minh City
ICU: Intensive care unit
IQR: Interquartile range
LMIC: Low- and middle-income country
LRTI: Lower respiratory tract infection
mAb: Monoclonal antibody
NCPAP: Nasal continuous positive airway pressure
NHIS: National health insurance system
RDA: Rapid detection of antigen
RSV: Respiratory syncytial virus
RT-PCR: Reverse transcription polymerase chain reaction
