# Supplementary material for: Estimating the economic burden of respiratory syncytial virus infections in infants in Vietnam: a cohort study
Source: BMC Infect Dis. 2023 Feb 6;23:73. doi: 10.1186/s12879-023-08024-2 (PMC9901829; doi:10.1186/s12879-023-08024-2)
Supplement: Supplementary file 1 — Additional file 1. Additional Tables. [file 12879_2023_8024_MOESM1_ESM.pdf]

Table S1. Demographic and clinical characteristics of patients having RSV RDA testing and those did not.

Characteristics	Inpatients at RD		
	Total (N=318)	RSV RDA tested (n=70)	RSV RDA not tested (n=248)
Age in months, median (IQR)	6 (2–11)	4 (2–11)	6 (2–11)
Number of females, n(%)	138 (43.5)	30 (42.9)	108 (43.6)
Number in HCMC, n(%)	123 (38.7)	17 (24.3)	106 (42.7)
Number of people living in household, median (IQR)	4 (4–5)	4 (4–5)	4 (4–5)
Number of children under 5 years old living in household, median (IQR)	1 (1–2)	1 (1–2)	1 (1–2)
Primary caregiver education of high school or higher, n (%)	199/314 (63.4)	43/70 (61.4)	156/244 (63.9)
Diagnosis at admission			
Bronchiolitis, n (%)	24 (7.6)	4 (5.7)	20 (8.1)
Pneumonia, n (%)	106 (33.3)	32 (45.7)	74 (29.8)
Other LRTIs, n (%)	187 (58.8)	34 (48.6)	153 (61.7)
Oxygen saturation (SaO_2)*			
SaO_2 < 90%, n (%)	3 (0.9)	0(0)	3 (1.2)
SaO_2 90–92	5 (1.6)	2 (2.9)	3 (1.2)
SaO_2 93 or above	310 (97.5)	30 (42.9)	148 (60.2)
Duration of hospitalization in days, median (IQR)	7 (6–9)	7.5 (6–9)	7 (6–8)
RSV positivity, n (%)	84	17 (24.3)	67 (27.0)

* *Oxygen saturation (SaO_2) was measured at admission as a marker of the severity of LRTIs (see method section).*

Abbreviations: HCMC, Ho Chi Minh City; ICU, intensive care unit; IQR, interquartile range; LRTI, lower respiratory tract infection; RD, Respiratory Department; RSV, respiratory syncytial virus.

Table S2. Cost associated with the societal perspective between patients having RDA test and those did not (median, IQR), in 2022 US dollars.

Cost components	RSV RDA testing		p value[1]
	RDA tested (n=70)	RDA non-tested (n=248)	
**Total costs**	216 (136 – 334)	177 (104 – 277)	0.009
Direct medical costs	102 (69 – 154)	81 (48 – 128)	0.02
Consultation fee	3 (2 – 4)	3 (2 – 6)	0.85
Medication costs	7 (5 – 18)	9 (4 – 20)	0.71
Laboratory costs	4 (2 – 15)	2 (2 – 13)	0.10
Procedure & service cost	59 (34 – 90)	39 (24 – 68)	0.01
Imaging costs	13 (3 – 19)	3 (3 – 16)	0.001
Miscellaneous costs*	16 (9 – 25)	12 (6 – 22)	0.10
Direct non-medical costs	43 (4 – 94)	32 (3 – 86)	0.22
Transport costs	9 (2 – 30)	6 (1 – 27)	0.53
Meal costs	67 (34 – 93)	19 (0 – 54)	<0.001
Accommodation costs	19 (0 – 36)	0 (0 – 26)	0.06
Caretaker costs	34 (34 – 34)	0 (0 – 41)	0.49
Indirect costs	28 (0 – 73)	18 (0 – 68)	0.21
Lost income	23 (0 – 71)	13 (0 – 67)	0.30
Lost leisure	3 (2 – 6)	2 (1 – 6)	0.51
Prior to hospital costs	16 (9 – 24)	17 (9 – 38)	0.68
In-hospital costs	215 (124 – 298)	154 (85 – 253)	0.003
Follow-up visit costs	5 (0 – 11)	0 (0 – 9)	0.02

** Miscellaneous costs include medical consumables such as syringes, gloves, and soap.*
[1] Mann-Whitney U p-value comparing between the costs associated with patients having RDA test and those did not.
Abbreviations: ICU, intensive care unit; IQR, interquartile range; RDW, RD, Respiratory Department; RSV, respiratory syncytial virus.

Table S3. Household-level out-of-pocket costs and payment sources between patients having RDA test and those did not (median, IQR), in 2022 US dollars.

Cost components	RSV RDA testing		p value[1]
	RDA tested (n=70)	RDA non-tested (n=248)	
**Total out-of-pocket costs**	**125 (64 – 208)**	**109 (55 – 184)**	**0.17**
(1) Direct medical costs	***23 (13 – 55)***	***24 (13 – 47)***	***0.82***
Consultation fee	3 (2 – 4)	3 (2 – 6)	0.85
Medication costs	2 (0 – 6)	3 (0 – 9)	0.03
Laboratory costs	0 (0 – 2)	1 (0 – 2)	0.41
Procedure & service cost	0 (0 – 17)	4 (0 – 16)	0.26
Imaging costs	0 (0 – 2)	1 (0 – 3)	0.09
Miscellaneous costs*	13 (4 – 20)	9 (2 – 17)	0.35
(2) Direct non-medical costs	43 (4 – 94)	32 (3 – 86)	0.22
Transport costs	9 (2 – 30)	6 (1 – 27)	0.53
Meal costs	67 (34 – 93)	19 (0 – 54)	<0.001
Accommodation costs	19 (0 – 36)	0 (0 – 26)	0.06
Caretaker costs	34 (34 – 34)	0 (0 – 41)	0.49
(3) Indirect costs	28 (0 – 73)	18 (0 – 68)	0.21
Lost income**	23 (0 – 71)	13 (0 – 67)	0.30
Lost leisure**	3 (2 – 6)	2 (1 – 6)	0.51
**Family response**			
Reducing other expenses	70.0%	55.7%	0.03
Use personal savings	88.6%	86.9%	0.56
Borrowed	31.4%	25.4%	0.31
Selling assets	7.1%	5.2%	0.54
Donations	22.9%	14.5%	0.09
Other	0%	0.4%	0.59

Note: Total out-of-pocket costs = sum of (1–3). These costs were incurred prior to, during, and after hospitalization. (2) and (3) are the same as costs associated with the societal perspective in Supp.Table 2.

** Miscellaneous costs include medical consumables such as syringes, gloves, and soap.*
*** Opportunity costs of income lost due to missed work were based on the average salary reported by participants in the study, and opportunity costs of lost leisure time were based on the minimum Vietnamese income based on government data for the study period* (18).
[1] Mann-Whitney U p-value comparing between the costs associated with patients having RDA test and those did not.
Abbreviations: ICU, intensive care unit; IQR, interquartile range; RD, Respiratory Department; RSV, respiratory syncytial virus.

Table S4. Detailed breakdown of direct medical cost components between patients having RDA test and those did not (median, IQR), in 2022 US dollars.

Cost components	RSV RDA testing		p value[1]
	RDA tested (n=70)	RDA non-tested (n=248)	
**Total direct medical costs**	102 (69 – 154)	81 (48 – 128)	0.02
Medication costs	7 (5 – 18)	9 (4 – 20)	0.71
Antibiotics	3 (1 – 6)	1 (1 – 4)	0.008
Corticosteroids	0.02 (0.02 – 0.04)	0.04 (0.03 – 0.11)	0.23
Bronchodilator oral	1 (0 – 1)	1 (1 – 1)	0.15
Bronchodilator inhalation	3 (2 – 8)	8 (2 – 18)	0.32
Other drugs	3 (2 – 5)	2 (2 – 4)	0.05
Laboratory costs	4 (2 – 15)	2 (2 – 13)	0.10
Imaging costs	13 (3 – 19)	3 (3 – 16)	0.001
Chest x-ray	3 (3 – 6)	3 (3 – 5)	0.12
Echocardiogram	-	7 (1 – 12)	N/A
Ultrasound	13 (10 – 13)	10 (2 – 13)	0.001
Procedure & service costs	59 (34 – 90)	39 (24 – 68)	0.01
Consultation costs	3 (2 – 4)	3 (2 – 6)	0.85
Miscellaneous costs	16 (9 – 25)	12 (6 – 22)	0.09

** Miscellaneous costs include medical consumables such as syringes, gloves, and soap.*
[1] Mann-Whitney U p-value comparing between the costs associated with patients having RDA test and those did not.
Abbreviations: ICU, intensive care unit; IQR, interquartile range; RD, Respiratory Department; RSV, respiratory syncytial virus.
